# Targeting NR4A Nuclear Receptors to Control Stromal Cell Inflammation, Metabolism, Angiogenesis, and Tumorigenesis

**DOI:** 10.3389/fcell.2021.589770

**Published:** 2021-02-09

**Authors:** Daniel Crean, Evelyn P. Murphy

**Affiliations:** ^1^School of Veterinary Medicine, University College Dublin, Dublin, Ireland; ^2^School of Medicine, University of Limerick, Limerick, Ireland

**Keywords:** NR4A nuclear receptors, inflammatory homeostasis, fibroblast-like and mesenchymal stromal cells, angiogenesis, cancer immunity and tumorigenesis, NR4A control of tumor-stromal communication

## Abstract

The NR4A1–NR4A3 (Nur77, Nurr1, and Nor-1) subfamily of nuclear receptors is a group of immediate early genes induced by a pleiotropy of stimuli including peptide hormones, growth factors, cytokines, inflammatory, and physiological stimuli, and cellular stress. NR4A receptors function as potent sensors of changes in the cellular microenvironment to control physiological and pathological processes through genomic and non-genomic actions. NR4A receptors control metabolism and cardiovascular and neurological functions and mediate immune cell homeostasis in inflammation and cancer. This receptor subfamily is increasingly recognized as an important molecular connection between chronic inflammation, altered immune cell responses, and cancer development. In this review, we examine how transcriptome analysis identified NR4A1/NR4A2 receptors as transcriptional regulators in mesenchymal stromal cell (MSC) migration, cell cycle progression, and cytokine production to control local immune responses. In chronic inflammatory conditions, such as rheumatoid arthritis, NR4A receptors have been shown to modify the activity of MSC and fibroblast-like stromal cells to regulate synovial tissue hyperplasia, pathological angiogenesis, and cartilage turnover *in vivo*. Additionally, as NR4A1 has been observed as a major transcriptional regulator in tumor–stromal communication controlling tumorigenesis, we discuss how advances in the pharmacological control of these receptors lead to important new mechanistic insights into understanding the role of the tumor microenvironment in health and disease.

## NR4A Subfamily of Nuclear Receptors

The NR4A1–NR4A3 (Nur77/NGFI-β/TR3, Nurr1/TINUR/NOT, and Nor-1/MINOR/CHN) subfamily of nuclear receptors is a group of immediate early genes induced by a pleiotropy of stimuli including peptide hormones, growth factors, cytokines, inflammatory and physiological stimuli, and cellular stress. NR4A receptors function as potent sensors of changes in the cellular microenvironment to control physiological and pathological processes through genomic and non-genomic actions. NR4A receptors control metabolism, vascular homeostasis, and cardiovascular and neurological functions and mediate immune cell homeostasis in inflammation and cancer. The majority of NR4A activity is due to direct activation or repression of transcriptional target expression. A growing body of information demonstrates direct non-genomic roles of NR4As through posttranslational modifications and interactions with binding partners (recently reviewed by Herring et al., 2019). NR4A receptors bind directly as monomers or homodimers to promoter regions of target genes that contain the NBRE response element (AAAGGTCA). Furthermore, NR4A receptors can dimerize with retinoid X receptor (RXR) nuclear receptors and bind to a DR5 (direct repeat with 5-bp length spacer) motif, providing a mechanism for NR4A receptors to regulate distinct target genes *in vivo*. Similar to NR4A receptors, RXR is also a member of the nuclear receptor subfamily controlling pathways associated with cell development, differentiation, metabolism, and cell death (Evans and Mangelsdorf, 2014).

Expression levels, subcellular localization, and the cellular context/environment can influence NR4A tissue-specific functional roles as observed in several cancers and diseases characterized by chronic inflammation (Zhao and Bruemmer, 2010; Mohan et al., 2012; Murphy and Crean, 2015). The cross talk of the NR4A1–NR4A3 subfamily members with various oncogene and tumor suppressor pathways is well-established (Safe et al., 2014; Beard et al., 2015; Wan et al., 2020). Extensive numbers of studies indicate that NR4A genes can act paradoxically either as oncogenes or as tumor suppressors. For example, they serve as oncogenes in lung cancer, melanoma, and colorectal cancer (CRC) but display tumor suppressor roles in acute myeloid leukemia (AML), breast cancer, and metastatic ovarian cancer. The NR4A1–NR4A3 cross talk with pro-tumorigenic or tumor-suppressive signaling pathways underwrites a dichotomous and context-dependent role(s) for members of this receptor subfamily (reviewed by Beard et al., 2015, and Wan et al., 2020).

Disordered stromal cell function, including altered signaling and secretion of paracrine factors, has been linked to disease progression of cancer and inflammatory disorders (Spaeth et al., 2008; Nissen et al., 2019). The molecular cross talk between cancer cells and their surrounding stroma and tumor microenvironment (TME) has a crucial role in the regulation of tumorigenesis and disease advancement. Evidence on the association between NR4A1–NR4A3 functional activities, their influence on the tumor–stromal cell environment, and the impact on disease pathogenesis is emerging. Innovative studies are providing compelling support that NR4A orphan receptors, by changing the local cellular microenvironment, can mediate inflammatory responses, promote angiogenesis, control cellular metabolism and survival, and alter cancer cell immunity to influence the course and outcome of disease. The purpose of this review is to discuss how recent advances in NR4A nuclear receptor biology are leading to important new mechanistic insights into understanding their role in stromal cells and the TME in health and disease.

## TME and Tumor Stroma

The TME is composed of non-malignant host cellular and non-cellular components, including cells of the immune system, blood cells, endothelial cells, fat cells, and the stroma. The tumor stroma is an essential component of the TME and is composed of cellular and non-cellular connective tissues that support functional tissues, including fibroblasts, mesenchymal stromal cells (MSCs), osteoblasts, chondrocytes, and the extracellular matrix (ECM). While specialized cells of the TME, such as endothelial cells, pericytes, adipocytes, and immune cells, can be included as constituents of the stromal compartment, these cells are more accurately defined as non-stromal cells within the TME (Valkenburg et al., 2018).

It is well-established that the TME plays a pivotal role in tumor progression, and together with cells of the stroma, this milieu plays a major role in maladapting and promoting tumor survival and progression. As outlined, the TME consists of a complex system of heterogeneous cancer cells alongside local host cells and non-stromal components of the TME including adipocytes, pericytes, and immune cells, which also contribute to the tumor–stromal communication (de Groot et al., 2017). Within this environment are fluxes in multiple signaling molecules such as cytokines and adenosine, alterations in metabolic states, regions of hypoxia, acidosis, fibrosis, structural/architectural breakdown from factors such as matrix metalloproteinases (MMPs), and enhanced angiogenesis concurrent with inadequate perfusion, leading to poor nutrient supply (Liotta and Kohn, 2001; De Palma et al., 2017; Binnewies et al., 2018; Chandler et al., 2019). This complex environment is not only a consequence of cancer progression but also a pivotal factor in aiding its progression (Liotta and Kohn, 2001; Binnewies et al., 2018).

The tumor stroma, like its normal counterpart, is essential for tissue structure and remodeling. It is a vital component of the TME involved in tumorigenesis, cancer progression, and metastasis (Valkenburg et al., 2018). In addition to the main components of the stroma, including MSCs, osteoblasts, and the ECM, the tumor stroma also has a high abundance of cancer-associated fibroblasts (CAFs). While stromal cells display antitumor activities in normal conditions, in the background of such altered and harsh environments, stromal cells respond and undergo phenotypic changes and in turn play a pivotal role in influencing tumor progression (de Groot et al., 2017). One of the most notable maladaptive responses is the activation of the hypoxia inducible factor-1α (HIF-1α)-dependent gene transcription in hypoxic tumors, leading to metabolic changes and increased oxygen supply, which aid tumor survival and progression, leading to worse outcomes for patients (Semenza, 2010). Predictably, several of these tumor-associated alterations, or factors which promote or maintain the complexity of these transformed systems, are targets for cancer therapy design, for example, targeting tumor angiogenesis, reprogramming cellular metabolism, and adjusting inflammatory responses leading to altered immune cell populations, with the goal of correcting the environment, which aids in the survival of cells that promote tumorigenesis (Semenza, 2010; Naing et al., 2018; Wang et al., 2018). As we outline herein, leading reports elucidating the novel regulatory role of the NR4A1–NR4A3 receptors implicate these receptors as important mediators controlling these crucial cellular processes leading to changes in the TME and cancer progression.

## (i) NR4A1–NR4A3 Receptors, the Local Inflammatory Response, and Tumor Stromal Cells

Tumor–stromal communication plays an important role in not only cancer initiation but also the proliferation and metastasis of these cells into distant organs, potentially causing cancer recurrence. Inflammatory cytokines and growth factors released by MSC, CAFs, and tumor-associated macrophages (TAMs) initiate a signaling cascade and create a microenvironment conducive to tumorigenesis (Guo and Deng, 2018; Leuning et al., 2018). In 2014, Zhou and colleagues revealed a distinct mechanism by which the microenvironment stimulates breast cancer cell invasion and metastasis. With a genome-wide cDNA screen, it was identified that inflammation-induced NR4A1 (NR4A2 and NR4A3 also respond) is a critical factor for the activation of TGF-β/SMAD-mediated breast cancer cell migration, invasion, and metastasis *in vitro* and *in vivo*. Loss of NR4A1 inhibits TGF-β-induced epithelial-to-mesenchymal transition (EMT), revealing a novel mechanism by which the microenvironment stimulates breast cancer cell invasion and metastasis (Zhou et al., 2014).

Furthermore, a recent study proposes a far-reaching model (using serial single-cell RNA sequencing) of paracrine signaling mediated by the activity of cyclooxygenase (COX-2) enzyme activity and production of the proinflammatory mediator, prostaglandin E2 (PGE2), by prolactin receptor (PLR^+^)-expressing tumor cells (Zheng et al., 2019). In this *in vivo* model of tumorigenesis, stromal cell expression and activity of all three NR4A receptors are activated by the enhanced secretion of PGE2 by neighboring tumor cells. NR4A receptors rapidly heterodimerize with the RXR, and a stromal cell NR4A–RXR complex induces the transcription, production, and secretion of the peptide hormone prolactin. The secreted hormone feeds back to neighboring tumor cells, increasing their proliferation, leading to tumorigenesis. Importantly, it was found that induction of stromal NR4A expression by PGE2 selectively stimulates expression of NR4A receptors but not the RXR family (Zheng et al., 2019). COX-2, prolactin, and prolactin receptor show consistent differential expression in tumor and stromal compartments across several human cancers. The observed cellular paracrine cross talk may be important factors in the efficacy of the anti-inflammatory COX-2 inhibitors in cancer suppression.

The COX-2/PGE2/NR4A signaling findings complement previous studies where NR4A has been shown to be a regulator of stromal and immune cell functions (Murphy and Crean, 2015) and linked to the expression of prolactin expression in inflammatory joint disease (McCoy et al., 2015). Further, in rheumatoid arthritis (RA), psoriasis, and colon cancer, the NR4A subfamily has previously been singled out as a downstream effector of prostaglandin (PGE) signaling. PGE2 potently induces NR4A2 expression levels via a cAMP/PKA-dependent pathway (Holla et al., 2011; McMorrow and Murphy, 2011). Studies by Holla et al. (2011) suggest that the molecular cross talk between PGE2 and NR4A2 is central to controlling CRC survival mediated through the regulation of apoptosis by blocking cleavage of caspase-3, with NR4A2 playing a central role as a point of transcriptional integration coupling eicosanoid and metabolic pathways.

Chronic inflammation can generate an immunosuppressive microenvironment that allows advantages for cancer formation and progression. MSCs, and their secreted paracrine factors, can modulate inflammatory and immune responses (Fontaine et al., 2016). The immunosuppressive environment has been shown to be PGE2 regulated in several cancers (Wang and Dubois, 2018). Recent investigations into the adaptation of the leukemic mesenchymal microenvironment reveal a novel COX-2/PGE2-NR4A/WNT signaling axis, correlating chronic inflammation with changes in cellular metabolism, leading to reduced immune surveillance (Wu et al., 2018). Reduced secretion of prostaglandins by the mesenchymal inhibition of COX-2 led to decreased expression of NR4A receptors and regulatory T-cell (Treg) genes, FOXP3 and CTLA4, in the MSC-cocultured CD34^+^ cells. The significance of these findings highlights that upregulated NR4A-WNT/β-catenin signaling functions to attenuate antileukemic immunity by upregulating Tregs and blocking the production of leukemia-reactive CD8^+^ cytotoxic T lymphocytes.

Tregs, which prevent overt immune responses and autoimmunity, have been shown to accumulate aberrantly in some types of TMEs to suppress antitumor immunity and to sustain the establishment of an immunosuppressive environment. Impeding Treg-mediated immune tolerance is central when considering cancer immunotherapy. Mice lacking *NR4A1* and *NR4A2* genes, specifically in Treg cells, show resistance to tumor growth in transplantation models without exhibiting any serious systemic autoimmunity (Hibino et al., 2018). Treatment with a chemotherapeutic agent, camptothecin, together with a COX-2 inhibitor was found to inhibit induction and transcriptional activity of NR4A factors, and they synergistically display antitumor effects *in vivo* (Hibino et al., 2018). Thus, genetic inactivation or pharmacologic inhibition of NR4A receptors can unleash effector activities of CD8^+^ cytotoxic T cells and stimulate potent antitumor immune responses within the TME. Chen et al. (2019) further ascertained that an NFAT–NR4A axis controls the expression of several inhibitory receptors and that treatment of tumor-bearing mice with CAR-T cells, lacking all three NR4A receptors, results in tumor regression and prolonged survival. Collectively, these studies indicate translational and therapeutic implications in the development of effective TME anticancer therapies by modulating NR4A receptor function in tumor-infiltrating T cells.

It is thought that MSCs exhibit a gain of function which allows them to preferentially migrate toward tumor sites as they would to a wound (Spaeth et al., 2008). The molecular mechanisms behind this movement are still not fully elucidated but are becoming more recognized as important, owing to the fact that MSCs within the TME are often linked with poorer prognosis, augmenting angiogenesis, tumor formation, and metastasis (Karnoub et al., 2007). In breast cancer, for example, many studies have used the MDA-MB-231 cell line to examine the plausible chemotactic factors responsible for this movement (Dwyer et al., 2007; Lin et al., 2008). This MDA-MB-231 cell line models the most invasive and migratory triple-negative (TN) form of breast cancer (Lanning et al., 2017). Signaling molecules including SDF-1α, TGF-β, IL-6, PDGF, MCP-1, and cyclophilin B were identified as trophic factors associated with MSC recruitment, but it is proposed that they are not working alone and that additional signaling pathways, transcriptional mechanisms, and regulatory elements need to be ascertained to fully understand the complexity of the molecular processes involved. The NR4A family is associated with the migration of MSCs and their movement into tumor sites. In a study examining the difference in gene profiles between migratory and non-migratory MSCs, it materialized that, of the 12 genes which displayed differential expression, both *NR4A1* and *NR4A2* genes showed the highest expression change in the migratory variety in response to SDF-1α and PDGF, signifying a potential regulatory role for these transcription regulatory factors (Maijenburg et al., 2012). Enhanced *NR4A1* and *NR4A2* expression levels in MSCs lead to increased cytokine and growth factor production, suggesting that these receptors regulate migratory MSCs with the capacity to specifically modulate the local immune response.

## (ii) NR4A1–NR4A3 Receptors, the TME, and Stromal Cell Hyperplasia

One of the first indicators that normal cells may be switching to a cancerous phenotype is the initiation of hyperplasia and changes in metabolic activity, where the tissue of an organ is enlarged due to an abnormal increase in cell reproduction and proliferation (Dupont et al., 1993). Recent studies discerning the associations with stromal and non-stromal cells of the TME and NR4A1–NR4A3-dependent changes on cell density, metabolic activity, cell hyperplasia, survival, and invasion are described herein.

In breast cancer, the density of the breast tissue, “how many” stromal cells it has, has often been associated with increased cancer risk and poorer prognosis. A functional role for NR4A2 has been suggested by a study examining the mechanisms behind obesity-associated breast cancer risk (Ghosh et al., 2010). In human adipose stromal cells (ASCs), NR4A2 is established to be a key regulator of *aromatase* gene transcription. The function of *aromatase* is the enzymatic conversion of androgen to estrogen, which catalyzes the final and rate-limiting step in estrogen biosynthesis. This increase in *aromatase* activity and subsequently estrogen levels in adipose tissue is proposed to be one of the main causal factors in the substantial increased likelihood that women classified as obese will develop breast cancer (Bulun et al., 2005). Ghosh et al. hypothesized that with increased breast cell density, there is a concomitant decrease in the tumor-suppressive function of BRCA1 and NR4A receptors. When NR4A2 expression and activity are depleted in isolated human ASCs, there is a substantial increase in *aromatase* gene expression and, therefore, estrogen biosynthesis, increasing the likelihood of breast cancer occurrence (Ghosh et al., 2010).

As a major component in the breast cancer microenvironment, ASCs are capable of secreting large quantities of metabolic substrates, such as fatty acids, to establish a metabolic TME. NR4A1 has been identified as a regulator for fatty acid uptake in breast cancer, leading to restrained fatty acid metabolism and inhibiting breast cancer progression both *in vitro* and *in vivo*. This study reveals that NR4A1 binds and recruits a corepressor molecule to the promoter regions of CD36 (also known as FA translocase) and fatty acid-binding protein 4 (FABP4), leading to transcriptional suppression, which hampers fatty acid uptake, leading to the inhibition of cell proliferation and impeding tumor cell growth *in vivo* (Yang et al., 2020). In contrast, in a study of melanoma, NR4A1 contributes to the metabolic adaptation of melanoma cells by regulating fatty acid uptake and oxidation (Li et al., 2018). The NR4A1-dependent metabolic adaptation protects melanoma cells undergoing loss of attachment (LOA) to the ECM and supports the survival of circulating tumor cells in the circulation. As highlighted, eicosanoid signaling and fatty acid metabolism through PGE2-mediated upregulation of NR4A2 receptors in colon cancer cells have been established (Holla et al., 2011). The studies propose that COX-2-derived PGE2 potentially regulates an adaptive shift in metabolism and NR4A2 activity central to this process. NR4A2 transcriptional activity increases fatty acid oxidation by inducing the expression and activity of several enzymes central to the fatty acid metabolic pathway. Collectively, these studies indicate that alteration of NR4A activity may fine-tune an adaptive shift to energy utilization via fatty acid oxidation, a metabolic adjustment that is observed in several types of cancer.

The role of NR4A1 in the TME has been further recognized with *in vivo* studies of tumor metastatic spreading models in wild-type (NR4A1^+/+^) mice and NR4A1^−/−^ mice (Hanna et al., 2015; Li et al., 2017). Expression of host NR4A1 was identified as a critical factor in antitumor metastasis because, in the absence of NR4A1, metastatic spreading was greatly accelerated (Li et al., 2017). Furthermore, this study reveals two potential key mechanisms by which the absence of NR4A1 expression facilitates cancer cell invasion and metastasis. Lack of NR4A1 in TME macrophages promotes inflammatory cytokine TNF-α production, which stimulates cancer cells to undergo EMT and promotes invasive properties. In addition, lack of NR4A1 results in reduced levels of colony-stimulating factor-1 receptor (CSF-1R) expression, which decreases the migratory capacity of inflammatory cells and subsequently hinders cell chemotaxis and tumor infiltration. These results unveil a novel function of NR4A1 in regulating tumor invasion and metastasis, which is consistent with a previous study reporting that NR4A1-deficient mice specifically lack “patrolling monocytes,” resulting in increased cancer lung metastasis *in vivo* (Hanna et al., 2015).

NR4A1 protein expression is also decreased in the mouse basal-like mammary tumors during the tumor progression process and in a large proportion of human TN breast cancer (TNBC) tumors. The low expression of NR4A1 protein in human TNBC samples is associated with advanced tumor stage, lymph node metastasis, and disease recurrence. Expression of NR4A1 in TNBC MDA-MB-231 cells significantly inhibits the proliferation, viability, migration, and invasion of these cells in culture and *in vivo* and the growth and metastasis of these cell-derived tumors in mice (Wu et al., 2017). These results demonstrate that NR4A1 functions to inhibit the initiation and growth of the MDA-MB-231 cell-derived tumors in mice by reducing their proliferation rate.

Constitutive migration and TGFβ-induced migration of breast cancer cells are reliant on nuclear and extranuclear expression of NR4A1, respectively, and it has been shown that selective NR4A1 antagonists inhibit both pathways by decreasing the NR4A1-dependent expression of β1-integrin, inhibit TGFβ-induced nuclear export of NR4A1, and induced EMT (Hedrick et al., 2016). It has been recognized in CRC tissues that aberrant NR4A1 expression in cancer tissues and cells of the TME acts to promote cell growth and survival by serving as an important mediator of the WNT/β-catenin and AP-1 signaling pathways (Wu et al., 2011). Moreover, the receptor can promote metastasis and invasion through controlling the MMP9/E-cadherin axis (Wang et al., 2014) and β1-integrin (Hedrick et al., 2017) and forming a feedforward loop with β-catenin under hypoxic conditions (To et al., 2014). These studies propose a novel molecular basis for understanding the biological properties of tumorigenesis and reveal that selective targeting of NR4A1 functional activity may represent a mechanism for altering cancer metastasis *in vivo*.

## (iii) NR4A Expression and Activity in Tumor Angiogenesis

Angiogenesis is one of the key factors associated with cancer progression, with studies finding that the balancing levels of pro- and anti-angiogenic factors, including vascular endothelial growth factor (VEGF) levels, in tissue reflect the aggressiveness with which tumor cells spread. Additional cellular players involved in angiogenesis include CAFs (Kalluri, 2016; Santi et al., 2018). VEGF has been found to potently activate the expression and functional activity of all three NR4A members, and in NR4A1^−/−^ knockout mice, tumor growth, angiogenesis, and microvessel permeability are almost completely inhibited, further purporting a critical mode for these orphan receptors in tumorigenesis (Zeng et al., 2006). Recent studies strengthen this significant body of work, further demonstrating that NR4A1 is essential for VEGF-A-induced pathological angiogenesis, tumor growth, and metastasis *in vivo* (Ye et al., 2019; Chen et al., 2020). Extending previous findings that tumor growth was inhibited in NR4A1 knockout mouse models, metastasis of colorectal tumor was completely inhibited in NR4A^−/−^ mice. Tumor masses were increased by ~70% and decreased by ~40% in transgenic EC-NR4A1-S mice and EC-NR4A1-DN mice, in which the full length and a dominant negative mutant of NR4A1 were induced and specifically expressed in the mouse endothelium. In human disease, NR4A1 is highly expressed in the vasculature and tumor cells of human melanoma and CRC tissues, but not in normal tissues. Furthermore, tumor angiogenesis and modulation of genes associated with angiogenesis were critically reduced in tumor tissues treated with NR4A1 shRNAs and selective minigenes (Ye et al., 2019; Chen et al., 2020). Silencing endothelial NR4A1 inhibits the proliferation and migration of tumor cells, indicating that the NR4A1 receptor functions as a regulator of tumor growth and metastasis *in vivo*. Together, these studies demonstrate that NR4A1 is a prospective therapeutic target with translational potential for several human cancers by targeting the vasculature within the TME.

## (iv and v) CAFs, Intra-Tumoral Fibrosis, and NR4A Receptors

CAFs are the dominant cell type within the stroma of tumors. They orchestrate paracrine pro-tumorigenic signaling with adjacent tumor cells, thus exacerbating the hallmarks of cancer and accelerating tumor malignancy. In breast cancer, up to 80% of the normal fibroblasts in breast tissue acquire the CAF phenotype during cancer progression (Kalluri, 2016; Santi et al., 2018). Molecular insights into the transcriptional programs that enable the oncogenic function of CAFs are emerging (Chan et al., 2018). The complete human NR profile in CAFs from clinical cutaneous squamous cell carcinoma (SCC) biopsies has recently been accomplished (Chan et al., 2018). Interestingly, both NR4A2 and NR4A3 are significantly upregulated in microdissected CAFs (*n* = 10). A highly similar NR4A2/NR4A3 profile was observed between the microdissected CAFs and explanted CAFs, indicating that the NR profile of SCC CAFs is retained during *in vitro* culture. Pharmacological targeting of specific driver NRs in CAFs diminished SCC invasiveness, proliferation, drug resistance, energy metabolism, and oxidative stress status.

Tumors have often been described as “wounds that do not heal,” and as such, it is not unforeseen that fibrosis not only is a major consequence of progressing tumors but also plays a causal role in their progression (Chandler et al., 2019; Chen and Song, 2019). It is CAFs within the stroma that are the main protagonists of such fibrosis in cancers, while other cells, such as immune cells, may also contribute in secreting fibrotic activators (Yamauchi et al., 2018). To date, the specific cell origin of these CAFs remains unknown, with the likelihood that they are derived from diverse cell types within the stroma (Cirri and Chiarugi, 2011; Kalluri, 2016). Interestingly, several factors involved in fibrosis are similarly implicated in multiple stages of cancer progression. Numerous ECM proteins, including fibronectin, are secreted by CAFs and have been shown to enhance tumor aggression, invasion, and metastasis. TGFβ, a major pro-fibrotic factor, has been shown to drive the differentiation of fibroblasts to CAFs, which aid in tumor aggression and invasiveness (Kalluri, 2016). Additionally, TGFβ receptor activation is responsible for the secretion of multiple MMPs within tumors, which play pivotal roles in tissue breakdown enhancing the metastatic capabilities (Hawinkels et al., 2014). Of note, activation of NR4A1 in breast cancer enhances TGFβ3 signaling, potentiating its oncogenic activities, by inducing SMAD7 degradation. Moreover, NR4A1 was shown to enhance TGFβ-induced EMT, a process later shown to be dependent on NR4A1 nuclear export. These studies confirm a pivotal role for NR4A1 in mediating TGFβ/p38-dependent induction of β-catenin in TNBC migration and invasion (Hedrick et al., 2016, 2017; Hedrick and Safe, 2017).

In a separate analysis, inflammatory cytokines, IL-1β, and TNF-α potently induce NR4A2 expression, which, in the presence of TGFβ, potentiates SMAD activation of fibrosis and cancer development (Zhou et al., 2014; Palumbo-Zerr et al., 2015). These responses are significantly amplified when NR4A is overexpressed, leading to TGFβ/SMAD-induced EMT and invasion by interacting with and promoting AXIN2-RNF12/ARKADIA-induced SMAD7 degradation to enhance the expression of activated TGFβ type I receptor (TβRI). These observations suggest that both NR4A1 and NR4A2 are important mediators in responding to inflammatory stimuli by activating TGFβ signaling and underline the need for further identification of NR4A1–NR4A3-specific functions in controlling intra-tumoral fibrotic pathways and the impact on EMT.

The functional activity of NR4A3 in breast and lung cancer progression has been examined, and a notable association between this NR4A family member and the tumor suppressor p53 was determined (Fedorova et al., 2019). *NR4A3* gene expression is a direct transcriptional target of p53, suggesting that the NR4A3 functional activity and tumor suppressor role in cancer progression is activated by p53. As part of this study, patient survival analysis, using a publicly available clinical data repository, was conducted, establishing that high levels of NR4A3 expression positively correlate with increased survival rates for patients with breast and lung cancers. Thus, these studies reveal that *NR4A3* is a novel transcriptional target of p53, which triggers apoptosis and has a tumor-suppressive role in breast and lung cancers (Fedorova et al., 2019). Whether NR4A3 is a *bona fide* tumor suppressor needs further elucidation. However, several lines of evidence support this notion. In *NR4A1*/*NR4A3* double-knockout mice, it was reported that loss of these two genes can result in the development of AML, due to uncontrolled expansion of myeloid progenitor cells (Boudreaux et al., 2012, 2019).

## (vi) NR4A1-NR4A3 receptors in rheumatic diseases

NR4A1–NR4A3 expression is elevated in synovial tissue MSC and fibroblast-like stromal cells (FLSs), macrophage, endothelium, cartilage, and PGE2-stimulated chondrocytes from patients with RA, psoriatic arthritis, or osteoarthritis, making NR4A receptors attractive targets in rheumatic and skin diseases (McMorrow and Murphy, 2011; Marzaioli et al., 2012; Shi et al., 2017; Xiong et al., 2020). Stromal cell hyperplasia and development of a “tumor-like pannus” are characteristics of chronic inflammatory diseases including RA and psoriatic arthritis (Bottini and Firestein, 2013). Expression of NR4A1/NR4A2 subfamily members are significantly upregulated in the pannus tissue and are mediators of cytokine, growth factor, and prostanoid (PGE2) action contributing to the hyperplastic and invasive phenotype of FLSs that leads to the modulation of MMP production and cartilage homeostasis (Mix et al., 2012).

## Potential for Targeting NR4A Receptors

NR4A receptors' expression and function in stromal cells and their influence on non-stromal cells of the TME are emerging as a promising area for the promotion of novel therapeutic targets in the treatment and prevention of human cancer progression. Developments on mechanisms of NR4A silencing and/or strategies for their activation are leading to new therapeutic interventions (Lee et al., 2014; Safe et al., 2014; Boudreaux et al., 2019). Over the last decade, multiple agents have been identified to modulate NR4A1–NR4A3 expression and functional activity *in vitro* and *in vivo* (Safe et al., 2014, 2016). While previously it was understood that NR4A receptors were not “druggable” targets given their bulky and inhospitable ligand binding domain (de Vera et al., 2019), endogenous mediators, including fatty acids, have been shown to act as potent modulators (de Vera et al., 2016, 2019). These modulating agents, both agonists and antagonists, have been examined extensively, with their binding capabilities and exact mechanisms of action being rationalized systematically (Safe et al., 2014, 2016; Munoz-Tello et al., 2020). More recently, Conneely et al. have made important therapeutic innovations in AML with the identification of several drugs that modulate NR4A1/NR4A3 expression and function (Boudreaux et al., 2019). Both NR4A1 and NR4A3 are silenced in AML and display tumor-suppressing qualities suppressing MYC activity. NR4A1/NR4A3 silencing in AML is mediated through the blockade of transcription elongation, while treatment with dihydroergotamine (DHE) rescued NR4A silencing and allowed their tumor-suppressing ability to reactivate with concomitant slowing of AML progression *in vivo* (Boudreaux et al., 2019).

**Figure 1:**
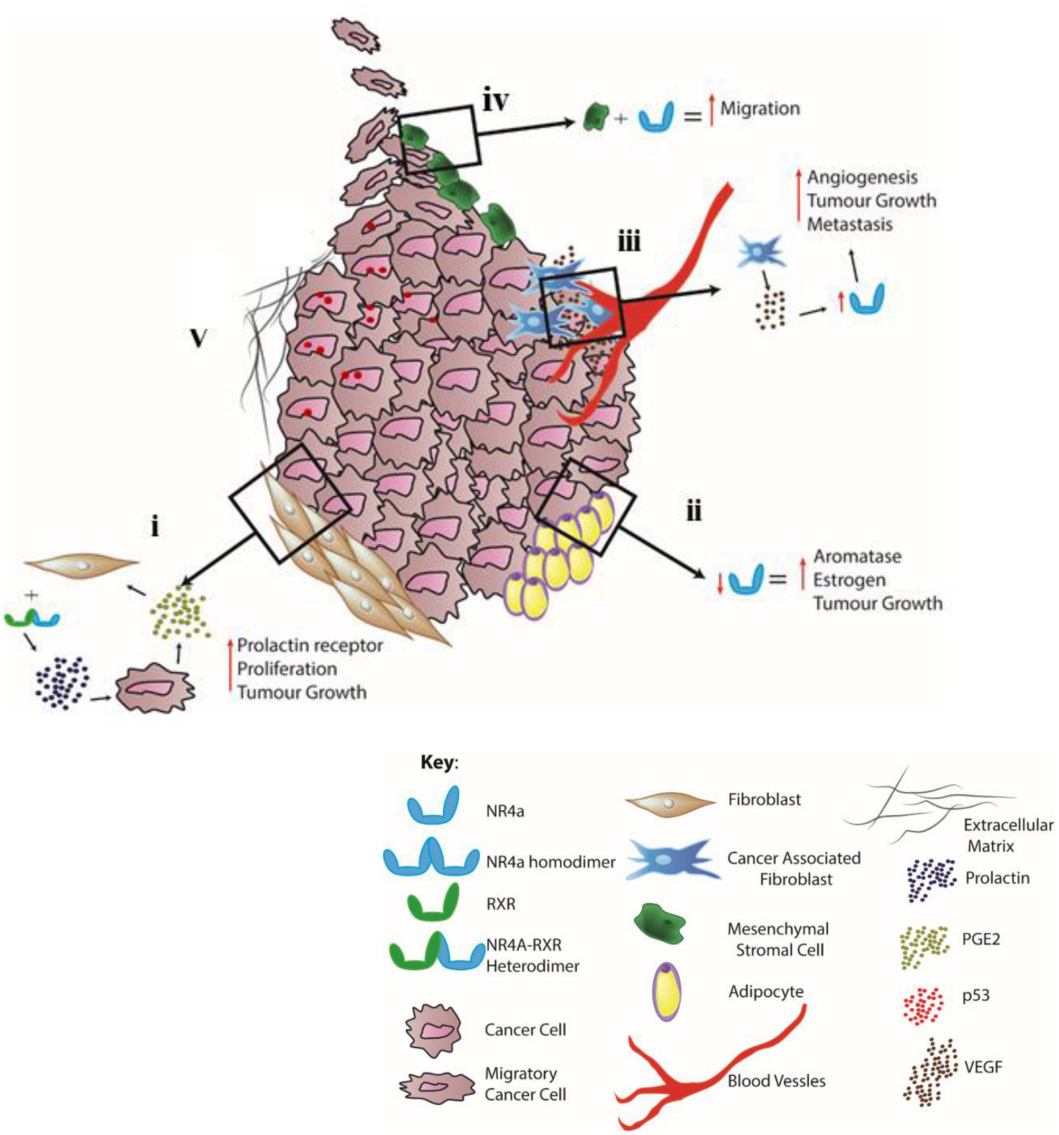
Distinct cell-specific functions controlled by NR4A1–NR4A3 nuclear receptors in the TME–stromal microenvironment. **(i)** Stromal fibroblasts: NR4A receptor dimerization with RXR receptors (downstream of pro-inflammatory COX-2/PGE2 activity) functions as potent regulators of prolactin production/signaling by stromal cells. **(ii)** Adipocytes: NR4A activity promotes an adaptive shift to energy utilization via fatty acid oxidation with NR4A2 regulating *aromatase* transcription and production of estrogen in TNBC. **(iii)** Endothelium and CAFs: NR4A receptors regulate VEGF-A-induced pathological/tumor angiogenesis, CAF activity, and tumor metastasis. **(iv)** MSC: NR4A1 functions in mediating MSC proliferation and cytokine and growth factor production, leading to cell migration resulting in altered immune (T regulatory, T cytotoxic, and macrophage) cell function. **(v)** ECM: cross talk between NR4As and the stroma, including interaction with the ECM, TGFβ/SMAD signaling, and p53 activity.

Thus, recent findings (summarized in Figure 1) support the targeting of NR4A receptors as potential specific therapeutic targets in enhancing the efficacy of cancer treatments and include (i) pursuing NR4A receptors (downstream of COX-2/PGE2 activity) as key regulators of prolactin production and signaling by stromal cells to reduce cancer cell proliferation and tumorigenesis; (ii) modulating NR4A2 transcriptional activity as a regulator of fatty acid utilization in CRC and *aromatase* transcription and production of estrogen in TNBC; (iii) controlling NR4A1–NR4A3 as regulators of angiogenesis, with the functional activity of NR4A1 in mediating VEGF-A-induced pathological/tumor angiogenesis facilitating cell proliferation and cancer cell migration; (iv) CAF activity, migration of MSCs and immune cells into tumor sites, and their role as mediators of EMT and cell metastasis; and (v) further analyzing the molecular interactions of this subfamily with the ECM, TGFβ/SMAD signaling, and control by altered p53 activity, which could also prove extremely beneficial to elucidating the NR4A1–NR4A3-dependent molecular mechanism(s) controlling cell survival, hyperplasia, and death and altering immune tolerance against cancer. Modulation of NR4A1–NR4A3 receptors as therapeutic targets in cancer biology reveals a novel category of mechanism-based therapies, and future efforts, due to their dichotomous responses, should involve meticulous evaluation of the cancer cell type targeted and moreover the method(s) of drug delivery, given the tissue- and cell-specific functional roles that NR4A receptors display in controlling cancer biology.

## Summary

While it is well-established that the initiation and development of cancer is a disease brought about by mutations in the cancer cells themselves, there is no doubt that the TME and stromal microenvironment are critically important for disease progression and tissue response to therapy. Recent developments discerning NR4A1–NR4A3 receptor function in cells within the tumor–stromal environment highlight the distinct functional role(s) that these receptors perform in modulating pro-inflammatory signaling, proliferation, hyperplasia, death/survival, migration, angiogenesis, and tumor immune surveillance. The described NR4A studies uncover the fundamental molecular mechanisms controlling cell-specific activity that facilitates tumor–stromal cell communication within the TME. With the development of mechanism-based NR4A biologics demonstrating efficacy at transforming tumor angiogenesis, growth, metastasis, and immune surveillance, these intracellular receptors may function as central receptors demonstrating translational capacity in cancer cell biology.

## Author Contributions

EM conceived the theme of the review. EM and DC wrote the manuscript and finally approved the manuscript.

## Conflict of Interest

The authors declare that the research was conducted in the absence of any commercial or financial relationships that could be construed as a potential conflict of interest.
